# Treatment of Hooping Cough

**Published:** 1853-01

**Authors:** 


*Treatment of Hooping Cough.*—In hooping cough, Dr. Madison, of Virginia, recommends a blister to the nucha; Dr. Golding Bird uses *conium*, in the following form:

℞ Ext. Conii, grs. xii.

Alum, grs. xxv.

Syrup Papaver, ʒiij.

Aq. fœniculi, ℥iij.

M.

A desert-spoonful every four or six hours.

Dr. Spengler, in a French journal, recommends *conia* in doses of one-fortieth of a grain for children three or four months old, and one-tenth of a grain for children of as many years. He has employed it in several cases successfully.—*Western Jour. of Med. and Surg.*

